# Another Year of Successes for PCD: Impact Factor, Collections, New Student Committee, Dr Lynne Wilcox Paper of the Year, and 2024 Calls for Papers

**DOI:** 10.5888/pcd20.230375

**Published:** 2023-11-16

**Authors:** Leonard Jack

**Affiliations:** 1 Preventing Chronic Disease, Office of Medicine and Science, National Center for Chronic Disease Prevention and Health Promotion, Centers for Disease Control and Prevention, Atlanta, Georgia

**Figure:**
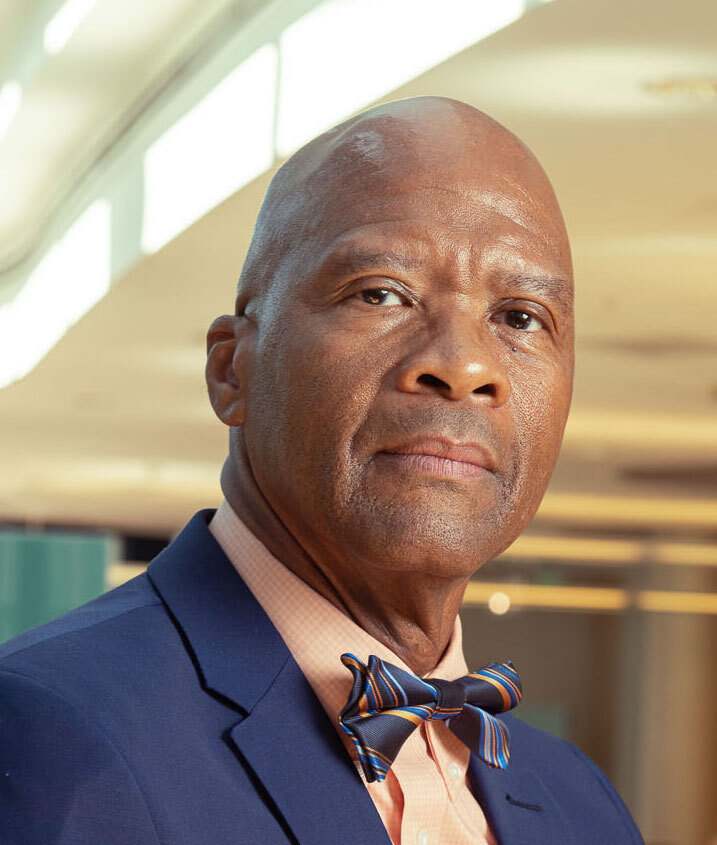
Leonard Jack Jr, PhD, MSc

*Preventing Chronic Disease* (PCD) brings 2023 to a close having achieved impressive accomplishments. This final Editor in Chief’s Column of the year provides updates on the journal’s increased impact factor; commitment to student development; publication of collections; formation of the Student Scientific Writing and Review Training Committee; upcoming 20th anniversary; and 2024 calls for papers. PCD has been positioned for success because of tremendous support from its editorial board, associate editors, Statistics Review Committee, and its pool of peer reviewers. PCD celebrates the hundreds of authors who submit articles annually to the journal for consideration. In addition, a major source of support to the journal has come from senior leadership in the National Center for Chronic Disease Prevention and Health Promotion (NCCDPHP).

## Impact Factor and Rankings

Over the past several years, PCD has continued to experience increases in the journal’s impact factor. The impact factor increased from 2.803 in 2020 to 4.354 in 2021 and 5.5 in 2022. In 2022, PCD was ranked third of 33 open-access US public health journals by Scientific Journal Ranking (SJR); 21st of 180 journals in SSCI Edition: Public, Environmental, and Occupational Health, Journal Citation Reports (JCR); and 59th of 608 journals worldwide in Public Health, Environmental, and Occupational Health by SJR. We are so very proud of this year’s impact factor and believe the journal’s momentum in publishing on critical and relevant topics in public health will lead to continued increases in the coming years. Clearly, the journal’s rankings indicate that its publication content resonates with readers around the world.

## 2023 Collections

PCD will publish 6 collections by the end of 2023 that address various public health topics. In June 2023, PCD released the collection *Combating Racism Through Research, Training, Practice, and Public Health Policies* (1). This collection consists of 8 articles that 1) capture data on exposure to racial discrimination and morbidity among diverse populations; 2) detail implementation of multicomponent antiracist initiatives enacted in schools of public health, schools of medicine, and other university-affiliated units; and 3) elevate attention to underlying drivers of structural inequities in housing and other domains through which meaningful community engagement in health initiatives is achievable.

In July, PCD released the collection *Health Equity in Action: Research, Evaluation, Policy, and Practice* featuring 11 articles that highlight the critical role that context, qualitative methods, and community-based participatory research play in efforts to achieve health equity (2). The collection covers topics such as the identification of resilient and at-risk neighborhoods for cardiovascular disease among Black residents; employment loss and food insecurity; using a health equity lens to reduce barriers to healthy food access; and the Centers for Disease Control and Prevention’s (CDC’s) guiding principles to promote an equity-centered approach to public health communication.

The bidirectional relationship between sleep and chronic disease has been underdiscussed in public health peer-reviewed literature. In August, PCD was excited to release its first-ever collection on the topic of sleep: *Sleep Deprivation, Sleep Disorders, and Chronic Disease*, which features 8 articles that discuss the interplay among sleep, mental health, and chronic disease, and emphasize the critical role that sleep plays in health outcomes and overall well-being (3).

State and local health departments serve as the backbone to the public health system in the US. PCD released a collection in September that highlights the roles of local and state health departments in helping to improve the nation’s health. Twenty-two articles were released in the collection *State and Local Health Departments: Research, Surveillance, and Evidence-Based Public Health Practices* (4). This collection offers examples of the impressive work in public health surveillance, epidemiology, geographic information systems, chronic disease prevention, and partnership development being led by state and local health department workforces (5). The collection is also dedicated in honor of Peter Briss, MD, MPH, for his unwavering support of PCD. Dr Briss is medical director of CDC’s NCCDPHP and director of its Office of Medicine and Science.

The collection *Implementation Evaluation: Assessing Efficiency, Effectiveness, and Impact of Public Health Programs* was released in early November (6). This collection consists of 10 articles showcasing peer-reviewed content that can be used by program planners, policy makers, evaluators, researchers, and other interested parties to refine evaluation methods, make health system improvements, strengthen collaborations and partnerships, build organizational infrastructure, measure return on investments, and enhance data collection approaches.

PCD’s final collection of the year, published in mid-November, is the *2023 Student Paper Contest*, which features 9 articles addressing several topics, including views about COVID-19 racial disparities in illness and death among community residents; effects of shared decision making on emergency department use among people with high blood pressure; the contribution of physical activity disparities to inequitable health-related quality of life among Black people with knee osteoarthritis; and dynamic patterns and modeling of COVID-19 early transmission (7). Camille Kroll and colleagues’ article “An Exploratory Qualitative Analysis of Explanations for COVID-19–Related Racial Disparities Among St. Louis Residents: ‘I Don’t Really Pay Attention to the Racial Stuff Very Much’ ” was selected as the winner in the master’s degree category (8). Donya Nemati and colleagues’ article “Investigating the Relationship Between Physical Activity Disparities and Health-Related Quality of Life Among Black People With Knee Osteoarthritis” was selected the winner in the doctoral category (9). PCD did not select winners in the high school, undergraduate, or postdoctoral categories. Congratulations to this year’s winners in the master’s and doctoral degree categories.

## Student Scientific Writing and Review Training Committee

PCD’s annual Student Paper Contest represents an example of the journal’s strong commitment to developing the future generation of public health researchers and evaluators. This year, PCD sought to build on this commitment by launching its first Student Scientific Writing and Review Training Committee (10). This committee consists of 17 students from across the United States. High school, undergraduate, graduate, and postgraduate students serve a 1-year appointment on the committee. During that time, students receive training on the following: 1) basic components of quantitative and qualitative research, 2) steps to critique and review publications, 3) effective scientific writing skills, 4) the peer review process conducted by journals, and 5) working with peers who bring different backgrounds, experiences, training, and interests to the table. Students also have an opportunity to hear from leading experts who share information on their training, experiences in public health research and evaluation, and current research and evaluation efforts. Students successfully meeting all requirements during the 1-year training will receive a certificate of completion.

## Dr Lynne Wilcox Paper of the Year

Positioning any journal for success requires being patient, having a well-developed plan of action, recruiting a strong pool of volunteers, committing to the dissemination of rigorously evaluated content that the public can trust, and demonstrating strong leadership. This year, PCD is especially proud to celebrate its many accomplishments and wants to recognize the many individuals who are responsible for the journal’s success. One example includes recognizing Dr Lynne Wilcox, PCD’s founding editor in chief, for her vision in establishing the journal 20 years ago. PCD made an important decision to recognize the substantial contribution Dr Wilcox made to nurture and grow a fledgling journal, and in her honor PCD will begin an annual award, the Dr Lynne Wilcox Paper of the Year. The Dr Lynne Wilcox Paper of the Year will be awarded to an author working in a local or state health department who serves as the first author of an article published in PCD. This award will recognize both Dr Wilcox’s contributions in establishing PCD and the work being led by local and state health departments.

## 2024 Calls for Papers

PCD remains committed to publishing content that advances the dissemination of the best evidence-based research, evaluation, and public health practice. In pursuit of this commitment, PCD draws readers’ attention to 3 active calls for papers. The first call for papers is for the collection *Comorbidity Causes, Health Implications, and Multisystem Approaches to Treatment and Care.* The journal is seeking submissions that facilitate a better understanding on how comorbidities can increase individual risk of complications or developing a new health condition altogether. Common comorbidities include obesity, COVID-19, high blood pressure, heart disease, diabetes, high blood lipid levels, arthritis, sleep apnea, depression, dementia, anxiety disorders, osteoarthritis, lung disease, and periodontal disease. The deadline to receive final manuscripts is February 15, 2024 (11).

PCD has an active call for papers for the upcoming collection *Public Health Nurse–Led Research, Practice, and Education in Disease Prevention and Control* (12). For this collection, PCD is seeking submissions about current public health nursing efforts to develop, implement, and evaluate the following: population-based interventions to prevent chronic diseases and control disease effects on quality of life, illness, and death; interventions that reduce the disproportionate incidence of chronic diseases among at-risk populations; and public health law and health policy–driven interventions. The deadline to receive final manuscripts is May 3, 2024.

The PCD 2024* Student Paper Contest* submission window remains open until Monday, March 25, 2024. PCD is interested in manuscripts authored by students serving as first author. Student manuscripts relevant to the prevention, screening, surveillance, and population-based intervention of chronic diseases, including but not limited to arthritis, asthma, cancer, depression, diabetes, obesity, cardiovascular disease, COVID-19, and other chronic conditions of interest to the journal (13).

## Conclusion

I conclude this year’s final Editor in Chief’s Column with a special thank you to Dr Peter Briss. The journal’s staff have reported to Dr Briss for 7 years, receiving his timely wisdom and guidance. He is retiring from CDC at the end of 2023 after 33 years of federal service. During his years with the journal he made concrete and lasting contributions to PCD’s success: identifying leading researchers, evaluators, and practitioners to serve as guest editors on collections; building PCD management capacity as the journal experienced growth; supporting the journal’s commitment to publishing rigorous content that the public could trust; identifying emerging areas in public health research, evaluation, and practice that require increasing attention in the published literature; expanding the journal’s focus on health systems research; and finding ways to support and honor PCD staff for their creativity, productivity, and innovation. In addition, he was an active participant in PCD’s editorial board meetings and in the journal’s first ever external review panel. The journal has benefited from his unwavering support, and thanks to his valuable leadership and expert contributions, PCD is well positioned for continued success. On behalf of the entire PCD staff, we congratulate Dr Briss on his stellar career in public health and express our sincere thanks for his leadership and advocacy of the journal.
